# A female with five chambers

**DOI:** 10.1007/s12471-019-01338-4

**Published:** 2019-10-25

**Authors:** K. K. Sahu, A. Doshi, A. K. Mishra, M. Kranis

**Affiliations:** 1 Department of Internal Medicine, Saint Vincent Hospital, 123 Summer Street, 01608 Worcester, MA United States; 2 Department of Cardiovascular diseases, Saint Vincent Hospital, 123 Summer Street, 01608 Worcester, MA United States

A 29-year-old female with a past medical history significant for intravenous drug abuse (IVDA), presented to the emergency room with complaints of fever, chills and shortness of breath. On initial assessment, patient was febrile (38.3 °C/101 F) and tachycardic (110/min). Auscultation revealed a pansystolic murmur most prominent at the right fourth intercostal space. Transoesophageal echocardiography (TEE) was done considering her high-risk behaviour and cardiac findings (Fig. 1). What is the diagnosis?

**Fig. 1:**
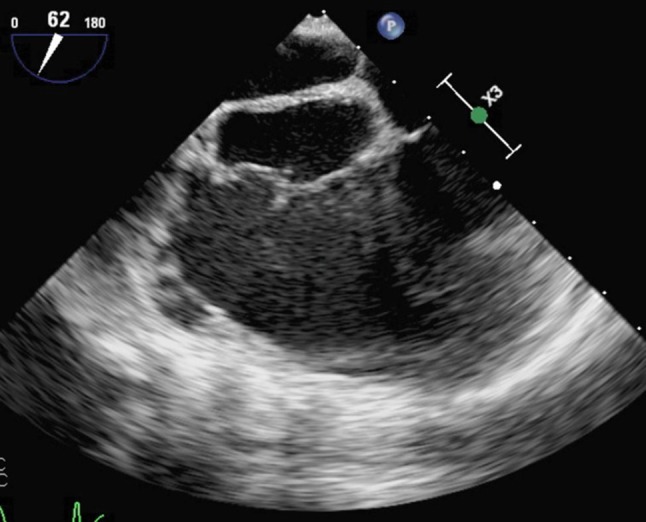
TEE (transoesophageal echocardiography) with a 4 chamber view

## Answer

You will find the answer elsewhere in this issue.

